# Development and validation of a nomogram for in-stent restenosis within 2 years in patients after iliac or femoral artery stent placement

**DOI:** 10.1186/s12893-025-02955-0

**Published:** 2025-05-21

**Authors:** YunSong Li, JiaTao Li, Yu Huang, Liang Li, PengKai Cao, XianChao Zhang, FengKai Wang, YaQi Wang, XiangDong Liu, YanRong Zhang

**Affiliations:** Department of Vascular Surgery, Hebei Medical University Third Hospital, 139 Ziqiang Road, Qiaoxi District, Shijiazhuang City, Hebei Province China

**Keywords:** Peripheral arterial disease, In-stent restenosis, Prediction model, Nomogram

## Abstract

**Objective:**

This study aimed to analyze the risk factors for in-stent restenosis (ISR) within 2 years after iliac or femoral artery stent placement.

**Methods:**

Clinical data of 237 patients diagnosed with peripheral arterial disease (PAD) and receiving iliac or femoral artery stent placement for the first time in the Third Hospital of Hebei Medical University from January 2015 to December 2022 were analyzed. Patients were randomized into training and validation set (7:3). Logistic regression was used to perform univariate and multivariate analysis on the possible factors of ISR, identify independent risk factors, establish a risk nomogram prediction model, and internally verify the predictability and accuracy of the model.

**Results:**

Binary logistic regression analysis showed that diabetes, hyperlipidemia, hyperfibrinogenemia and below-the-knee run-offs were independent risk factors for ISR within 2 years after iliac or femoral artery stent placement in patients with PAD. Based on these factors, the risk prediction model is established. The c index of the model was 0.856. The results showed that the risk prediction model has good accuracy in predicting ISR within 2 years after iliac or femoral artery stent placement.

**Conclusions:**

The risk prediction model based on the 4 risk factors of diabetes, hyperlipidemia, hyperfibrinogenemia and below-the-knee run-offs has good predictive performance.

## Introduction

Peripheral arterial disease (PAD) is a disease caused by the formation of atherosclerotic plaques in arteries of lower extremities, resulting in arterial stenosis or even occlusion, eventually leading to chronic limb ischemia. PAD incidence is increasing due to societal development, improved living standards, and aging demographics. A study has shown that the prevalence of PAD in the population aged 60–90 years in Sweden is 18% [1]. In China, the prevalence is 15.91% [2, 3].

The European Society of Cardiology guidelines recommend [3] that stent implantation is an important treatment for iliac or femoral artery lesions [1], but the occurrence of in-stent restenosis (ISR) seriously affects the long-term efficacy of endovascular treatment. The European Society for Vascular Surgery states that while most cases are amenable to endovascular or surgical revascularisation, maintaining long-term patency is often challenging. Early and late complications, as well as local and distant recurrences, frequently lead to failure of revascularisation [4]. The incidence of ISR within 2 years of implantation reported in Literature is nearly 30% to 40% of patients who undergo stent implantation for femoropopliteal disease [5]. Hence, tools for the identification of individual patients at higher risk for ISR with stent implantation are especially needed. Although previous studies have shown that the high incidence of ISR is related to vascular inflammation, neointimal hyperplasia and thrombosis, there are still some limitations in clinical application [6]. The identification of risk factors for ISR in these studies was limited to local vascular anatomical function and lacked assessment of patient history and clinical data, and therefore was not comprehensive enough. In addition, clinicians are unable to assess the probability of ISR after stenting on a patient-by-patient basis, and therefore are unable to accurately identify high-risk patients for the development of precisely targeted prevention programmes. In clinical diagnosis and management, most existing prediction tools are based on limited clinical indicators that are not systematic and comprehensive, making it difficult to accurately identify patients at high risk of ISR. Although some studies have attempted to construct prediction models, the sensitivity and specificity of the models are poor and their generalisability to different healthcare settings and patient groups is questionable. At present, we urgently need a solid, well-validated and easy-to-use clinical ISR prediction model to accurately predict the occurrence of ISR, early intervene, and reduce the occurrence of ISR.

Featured by the advantage of visualization, nomogram can assess straightforward the probability of disease for individual patients without complex formula. Because of its convenience and operability, it has become an important tool for clinicians to make clinical decisions.

Based on these premises, this study retrospectively analyzed the clinical data of patients diagnosed with PAD and receiving iliac or femoral artery stent placement for the first time in the Third Hospital of Hebei Medical University, and explored the risk factors of ISR within 2 years after iliac or femoral artery stent placement. These risk factors were used to develop and validate a nomogram prediction model to help clinicians identify high-risk ISR patients, optimize treatment strategy, and improve patients prognosis.

## Methods

### Study population

This study is a retrospective cohort study. According to the inclusion criteria, we reviewed collected 237 clinical data of PAD patients who have received iliac or femoral artery stent placement for the first time in the Third Hospital of Hebei Medical University from January 2015 to December 2022. Finally, We finished a chart review of all femoral and iliac stents placed during this time frame. According to local regulations, the protocol was approved by the Ethics Committee of the Third Hospital of Hebei Medical University. We confirmed that all methods were performed in accordance with the relevant guidelines and regulations. Since this is a retrospective study using existing research data, we obtained a waiver of informed consent from the Ethics Committee and obtained the approval of the Ethics Committee of the Third Hospital of Hebei Medical University. This study adhered to the Declaration of Helsinki.

**Table:** 

Inclusion criteria	Excluded criteria
Patients over the age of 40	Patient age less than 40 years
Patient's clinical data were complete	Patient's clinical data were incomplete
Computed Tomography Angiography (CTA) or Digital Subtraction Angiography (DSA) confirmed the presence of iliac or femoral artery lesions	CTA or DSA confirmed the presence of other artery lesions (For example, the brachial artery)
Patients received iliac or femoral artery stent placement for the first time and the patient has not undergone any other surgery	Patients who underwent arterial thrombectomy or catheter thrombolysis or endarterectomy or Patients were treated with drug-coated balloon (DCB) and drug-eluting stent;
Stent was successfully implanted in the iliac or femoral artery and completed coverage of the lesion	Patients received both iliac and femoral artery stent placement
Postoperative DSA confirmed that the stenosis of iliac or femoral artery was less than 30%	Postoperative DSA confirmed that the stenosis of iliac or femoral artery was more than 30%

Diagnostic criteria of PAD:

①The age of the patient is > 40 years old;

②Patients with smoking, diabetes, hypertension, hyperlipidemia and other high risk factors;

③The patient has symptoms of PAD, such as intermittent claudication, resting pain, etc.;

④The distal artery pulsation of the ischemic limb of the patient was disappeared;

⑤The patient's Ankle-Brachial Index (ABI) ≤ 0.9;

⑥Doppler ultrasound, CTA, Magnetic Resonance Angiography (MRA), DSA and other imaging examinations of the patient.

The sample size was estimated using the event per variable (EPV) method, and since the recommended empirical guideline in logistic regression is a sample size of 10–15 times the number of covariates, this study used EPV = 10, involving 15 covariates, so ≥ 150 patients were required [7]. Finally, 237 patients were included. DSA or CTA were used to assess the vascular status of all patients. The standard of ISR within 2 years was defined as the stenosis of iliac or femoral artery confirmed by DSA examination was greater than 50% [8]. Of the 237 patients, 61 patients developed ISR, and the restenosis rate was 25.74%.

### Risk factors

Based on clinical plausibility and previous studies, A total of 15 candidate factors were identified for further processing,including clinical characteristics (age, sex, hypertension, diabetes, hyperlipidemia, smoking), laboratory test (albumin, globulin, Albumin-to-Globulin Ratio (AGR) [9], Neutrophil-to-Lymphocyte Ratio (NLR) [10], Platelet-to-Lymphocyte Ratio (PLR) [11], creatinine,fibrinogen) and imaging characteristics (location of stents, below-the-knee run-offs, length of stent [12, 13]). Fibrinogen > 4 g/L is defined as hyperfibrinogenemia [14], hyperlipidemia was defined as Triglycerides (TG) > 150 mg/dl or Low-Density Lipoprotein Cholesterol (LDL-C) > 140 mg/dl or both [15]. AGR is defined as the ratio of albumin and globulin, NLR is defined as the ratio of absolute counts of neutrophils and lymphocytes within 3 days after surgery, PLR is defined as the ratio of absolute counts of platelet and lymphocytes within 3 days after surgery.

### Statistical analysis

By searching the medical record system of the Third Hospital of Hebei Medical University, we finally obtained 237 cases that met the inclusion criteria. In these cases, 61 patients developed ISR. All the data in this study were analyzed by R software (4.2.0). The data collected in this study was divided into training and validation sets according to the random number table method, and the ratio of sample size between the training and validation sets was 7:3. The binary variables were compared by chi-square test or fisher exact test, and the continuous variables were compared by t test or ranked sum test. Univariate and multivariate logistic regression analysis was used to analyze the risk factors of ISR within 2 years after iliac or femoral artery stent placement in the training set. No correction for multiple comparisons was made in this study. The independent variable *P *< 0.1 in univariate analysis was included in multivariate regression analysis. The logistic regression model with the least amount of information in the Akaike information criterion (AIC) was selected as the final prediction model and the R software is used to visualize the output through the nomogram. The Receiver Operating Characteristic (ROC) curve was plotted to assess discrimination and calibration of the model by the area under curve (AUC). The calibration curve was plotted to compare actual risk with predicted risk; The decision curve analysis (DCA) was plotted to evaluate the clinical application value of the nomogram by calculating the net benefit under different threshold probabilities. The DCA was performed on both the validation set and the training set.

## Results

### Patient characteristics

In this study, a total of 237 patients with PAD who underwent iliac or femoral artery stent placement were included in the study. 166 patients were randomly assigned to the training set and 71 patients were randomly assigned to the validation set. Table 1 lists 15 variables, including the following aspects: clinical characteristics, laboratory test and imaging characteristics. Of these, 61 patients (44 in the training set and 17 in the validation set) developed ISR. Comparisons between the training and validation set showed no significant differences in all ISR risk-related factors (all *P* > 0.05).

**Table 1:** Baseline characteristics of the training set and validation set

Factors	Training set (*n* = 166)	Validation set (*n* = 71)	P
Age (year)	67.21 ± 9.61	67.68 ± 10.21	0.74
Gender, n (%)			0.16
Male	133 (80.1%)	63 (88.7%)	
Female	33 (19.9%)	8 (11.3%)	
Smoke, n (%)			0.74
Yes	88 (53%)	40 (56.3%)	
No	78 (47%)	31 (43.7%)	
Hypertension, n (%)			0.90
Yes	113 (68.1%)	47 (66.2%)	
No	53 (31.9%)	24 (33.8%)	
Diabetes, n (%)			0.99
Yes	65 (39.2%)	27 (38%)	
No	101 (60.8%)	44 (62%)	
Hyperlipidemia, n (%)			0.78
Yes	68 (41%)	27 (38%)	
No	98 (59%)	44 (62%)	
Albumin,g/L	38.52 ± 4.55	38.31 ± 4.56	0.89
Globulin,g/L	24.96 ± 5.15	24.83 ± 4.22	0.95
AGR	1.61 ± 0.39	1.61 ± 0.35	1.00
NLR	4.51 ± 2.19	4.51 ± 2.54	0.58
PLR	189.39 ± 101.38	181.84 ± 84.79	0.88
Creatinine			0.27
> 81 μmol/L	30 (18.1%)	8 (11.3%)	
< 81 μmol/L	136 (81.9%)	63 (88.7%)	
Hyperfibrinogenemia			0.32
Yes	64 (38.6%)	33 (46.5%)	
No	102 (61.4%)	38 (53.5%)	
Below-the-knee run-offs			1.00
3	110 (66.3%)	47 (66.2%)	
< 3	56 (33.7%)	24 (33.8%)	
Location of stents			0.64
Femoral artery	82 (49.4%)	32 (45.1%)	
Iliac artery	84 (50.6%)	39 (54.9%)	
Length of stent (mm)	144.36 ± 8.22	157.32 ± 11.63	0.38

### Clinical indicator analysis

The results of univariate logistic regression analysis of the training set (Table 2) show that: the related factors (*P* < 0.1) of ISR within 2 years in patients after iliac or femoral artery stent placement were diabetes, hyperlipidemia, albumin, globulin, AGR, NLR, creatinine > 81 μmol/L, hyperfibrinogenemia, below-the-knee run-offs, location of stents.Multivariate logistic regression analysis showed that the independent risk factors (*P* < 0.05) were diabetes, hyperlipidemia, hyperfibrinogenemia, below-the-knee run-offs, as shown in Table 3.

**Table 2:** Univariate logistic regression analysis of risk factors of ISR within 2 years in patients after iliac or femoral artery stent placement in training set

Factors	ISR group (*n* = 44)	Non-ISR group (*n* = 122)	Wald value	OR value (95% CI)	P
Age (year)	66.05 ± 10.30	67.63 ± 9.36	0.88	0.98(0.95,1.01)	0.35
Gender			2.02	0.55(0.25,1.25)	0.16
Female	12(27.27%)	21(17.12%)			
Male	32(72.73%)	101(82.88%)			
Smoke			0.056	1.09(0.55,2.17)	0.81
Yes	24(54.54%)	64(52.46%)			
No	20(45.46%)	58(47.54%)			
Hypertension			0.60	1.35(0.63,2.89)	0.44
Yes	32(72.73%)	81(66.39%)			
No	12(27.27%)	41(33.61%)			
Diabetes			11.76	3.51(1.71,7.20)	0.001
Yes	27(61.36%)	38(31.15%)			
No	17(38.64%)	84(68.85%)			
Hyperlipidemia	24/20	44/78	4.48	2.13(1.06.4.28)	0.034
Yes	24(54.55%)	44(36.07%)			
No	20(45.45%)	78(63.93%)			
Albumin	36.97 ± 5.03	39.07 ± 4.25	6.62	0.90(0.84,0.98)	0.01
Globulin	26.64 ± 5.51	24.35 ± 4.90	6.15	1.09(1.02,1.17)	0.013
AGR	1.46 ± 0.40	1.66 ± 0.37	8.40	0.23(0.083,0.62)	0.004
NLR	5.41 ± 2.73	4.19 ± 1.87	8.93	1.27(1.09,1.49)	0.003
PLR	201.03 ± 106.97	185.19 ± 99.41	0.78	1.00(0.99,1.01)	0.38
Creatinine > 81 μmol/L			5.10	2.59(1.13,5.91)	0.024
Yes	13(29.55%)	17(13.93%)			
No	31(70.45%)	105(86.07%)			
Hyperfibrinogenemia			12.44	3.65(1.78,7.49)	0.00
Yes	27(61.36%)	37(30.33%)			
No	17(38.64%)	85(69.67%)			
Below-the-knee run-offs			34.25	0.092(0.041,0.20)	0.00
3	12(27.27%)	98(80.33%)			
< 3	32(72.73%)	24(19.67%)			
Location of stents			14.43	4.47(2.06,9.68)	0.00
Femoral	33(75%)	49(40.16%)			
Iliac	11(25%)	73(59.84%)			
Length of stent(mm)	150.23 ± 106.91	142.24 ± 105.87	0.19	1.00 (0.99,1.00)	0.67

**Table 3:** Multivariate logistic regression analysis of risk factors of ISR within 2 years in patients after iliac or femoral artery stent placement in training set

Factor	B	SE	Wald-χ	P	OR (95% CI)
Diabetes	0.947	0.447	4.499	0.034	2.579(1.075,6.189)
Hyperlipidemia	1.153	.461	6.266	0.012	3.168(1.284,7.812)
Hyperfibrinogenemia	1.235	.455	7.381	0.007	3.440(1.411,8.388)
Below-the-knee run-offs	−2.499	.463	29.190	0.00	0.082(0.033,0.203)

### Drawing and validation of a nomogram

**Fig. 1:**
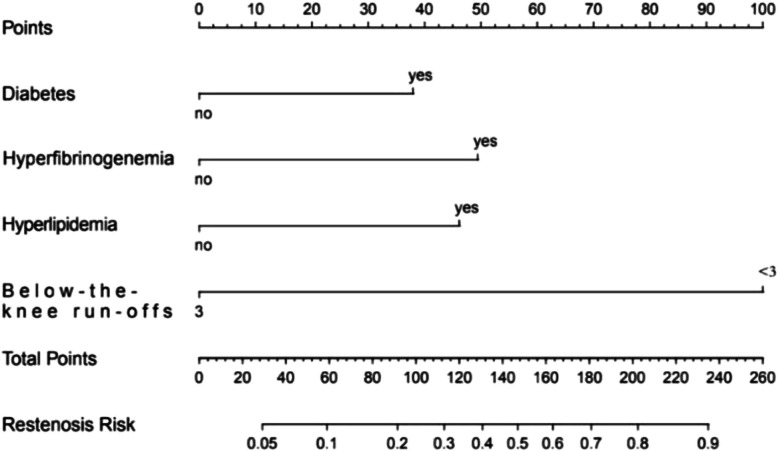
Nomogram for prediction of ISR within 2 years in patients after iliac of femoral artery stent placement

Based on univariate and multivariate logistic regression analysis, we obtained four independent risk factors and established a nomogram prediction risk model for ISR after iliac or femoral artery stent placement (Fig. 1).

Clinicians can use the nomogram to score patients. The nomogram contains four scoring indicators diabetes, hyperlipidemia, hyperfibrinogen and below-the-knee run-offs. The four scores are added together to obtain the final score, and the corresponding probability of ISR is found according to the points. By calculating the probability of patients developing ISR, identifying high-risk groups and carrying out early prevention, the incidence of ISR can be reduced.

**Fig. 2:**
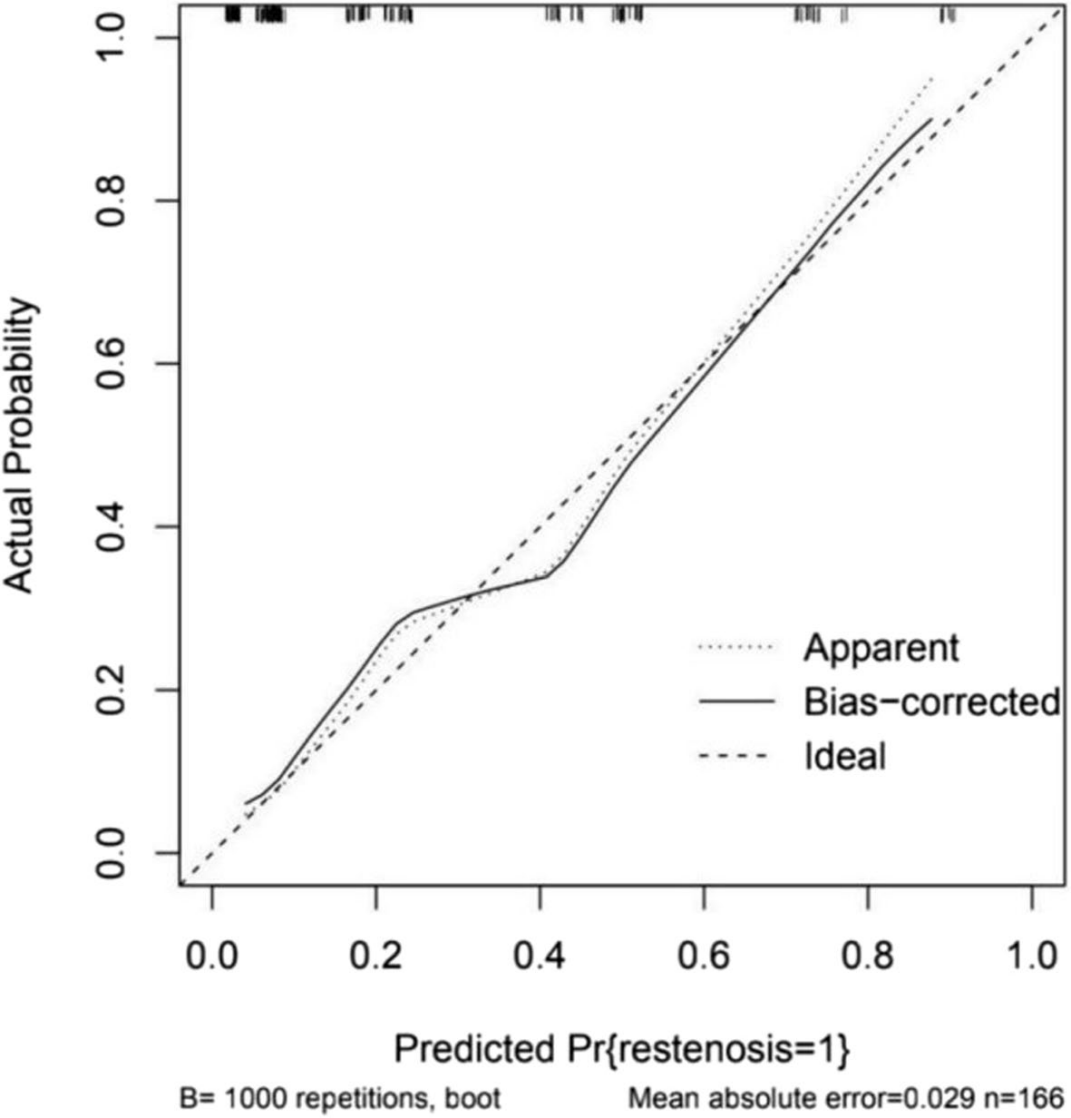
Calibration curve of nomogram model

**Fig. 3:**
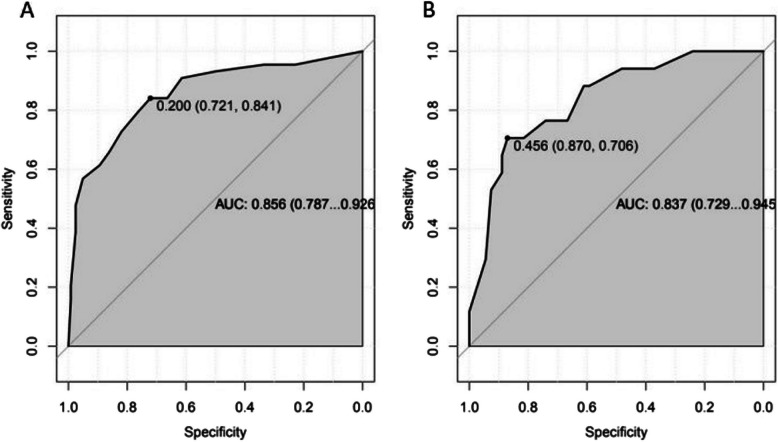
ROC curve of nomogram model for predicting the risk of ISR within 2 years in patients after iliac or femoral artery stent placement. **A** ROC curve of training set (AUC = 0.856); **B** ROC curve of validation set (AUC = 0.837)

**Fig. 4:**
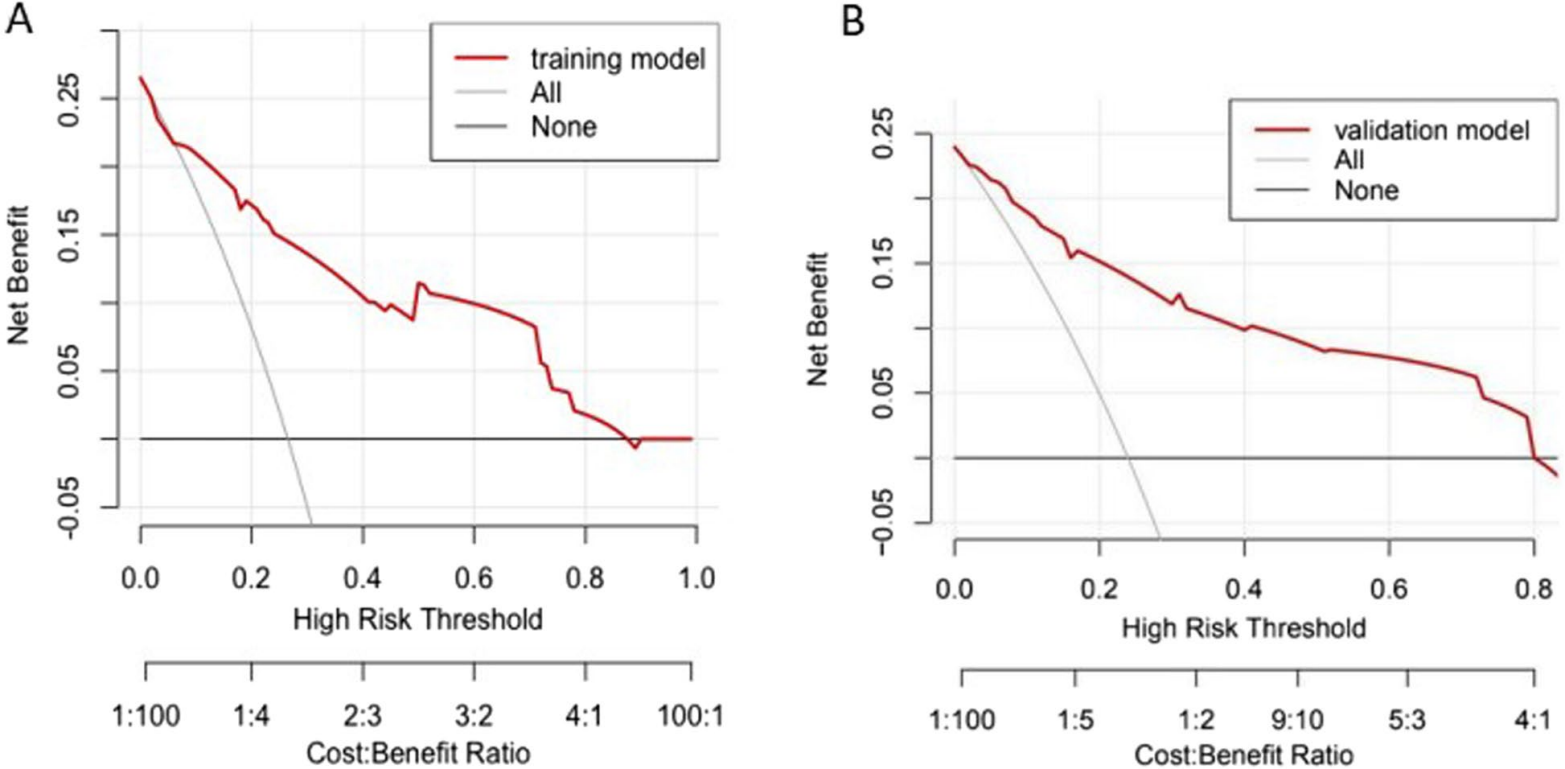
Decision curve of nomogram model for predicting the risk of ISR within 2 years in patients after iliac or femoral artery stent placement. **A** decision curve of training set; **B** Decision curve of validation set

The model was internally validated by the parallel Bootstrap method (after repeated sampling of the included data 1,000 times), and the calibration curve was close to the ideal curve (Fig. 2), indicating that the predicted ISR of the nomogram is highly consistent with the actual ISR. The ROC curves of the training set and the validation set drawn by the nomogram (Fig. 3) showed that the AUC of the training set was 0.856 (95% CI: 0.787–0.926), and the AUC of the validation set was 0.837 (95% CI: 0.729–0.945), indicating that demonstrates good discriminatory performance for ISR risk stratification and accuracy for the high-risk population of ISR. In this study, R language is used to calculate the best cut-off value according to Youden index, which is used to identify high-risk groups and provide clinical guidance. In the AUC of training set, the cut-off value is 0.200. In this value, the corresponding specificity value is 0.721, the corresponding sensitivity value is 0.841. Under the cut-off value implies low risk and over the cut-off value implies high risk. The clinical decision curve (Fig. 4) shows that using the nomogram to predict ISR can achieve greater benefits if threshold probability is < 87%. In this DCA curve, the x-coordinate represents the threshold probability and the y-coordinate represents the net benefit. At threshold probability < 87%, the prediction model was used to predict and intervene in patients placing femoral or iliac artery stent, resulting in a better clinical benefit. Therefore, the nomogram could be used to predict the risk of ISR within 2 years after femoral artery or iliac artery stent implantation which has a high accuracy and a wider range of threshold probabilities, and it might have potentially great significance in clinical application.

## Discussion

Stent implantation is an effective therapy for PAD. However, ISR seriously affects the long-term efficacy of endovascular treatment. According to the current research, the causes of ISR can be summarized as follows: 1) stent underexpansion [16]; 2) stent fracture; 3) vascular intimal hyperplasia; 4) in-stent neoatherosclerosis; 5) in-stent thrombosis [17]. Under the condition of successful stent implantation, this study mainly discusses the last three factors.

In this study, diabetes, hyperfibrinogenemia, hyperlipidemia, and below-the-knee run-offs were found to independently predict ISR within 2 years in patients with PAD who receiving iliac or femoral artery stent placement. Based on these independent risk factors, we established a risk nomogram prediction model that provides a solid, well-validated and easy-to-use tool for clinicians to conduct individualized assessment of patients at high risk of ISR. In addition, prospectively informing patients after stent implantation to control risk factors is of great significance for reducing the rate of ISR.

Diabetes is an important risk factor for coronary artery disease, stroke and PAD [18]. The risk for PAD is as much as three to four times higher in diabetic patients, and this risk increases by 26% with every 1% increase in the HbA1c level [19]. Similarly, diabetes is a factors for ISR. Endothelial dysfunction is one of the causes of PAD and ISR. Stent implantation can cause vascular endothelial injury, and in the environment of hyperglycemia, monocyte chemoattractant protein-1 (MCP1) and vascular cell adhesion molecule-1 (VCAM1) expression increased, enhance the interaction between monocytes and endothelial cells, and further promote endothelial dysfunction [20]. Endothelial dysfunction leads to reduced secretion of cytokines that inhibit the proliferation and migration of smooth muscle cells. Excessive proliferation and migration of smooth muscle cells is another important reason for ISR. In addition, endothelial dysfunction further promotes thrombosis, leading to the occurrence of ISR [21]. Diabetes also promotes platelet activation. As one of the markers of platelet activation, platelet-derived microparticle (PDMP) plays an important role in coagulation.

Diabetes patients typically display hypercoagulability and platelet hyperaggregability with increased levels of PDMP which suggests that patients with diabetes are more likely to develop ISR [22]. In an expert consensus about the prevention of cardiovascular diseases in people with diabetes, it is pointed out that exercise, including aerobic exercise, resistance exercise, and their combination, can not only control blood glucose, but also control the occurrence of cardiovascular disease [16]. In other words, controlling blood glucose can reduce the risk of ISR.

Fibrinogen is a new biomarker of oxidative stress and inflammation in recent years [23]. Hyperfibrinogenemia is an independent risk factor for cardiovascular and cerebrovascular diseases and ISR after coronary stent implantation [24, 25]. Fibrinogen is mainly expressed in hepatocytes, and its synthesis is regulated by inflammatory factors, such as IL-6 derived by monocytes, macrophages and vascular endothelial cells [26, 27]. In our study, Hyperfibrinogenemia suggests that the patient is in a hypercoagulable state and the risk of thrombosis is increased, which is one of the causes of ISR. In addition, fibrinogen is an important inflammatory marker that plays an important role in platelet aggregation [28]. After stent implantation, mechanical vascular injury occurred, which causing the aggregation of inflammatory cells and the production of cytokines. Fibrinogen also promotes endothelin-1 (ET-1) secretion by endothelial cells and ET-1 can effectively constricts blood vessels [29]. In addition, fibrinogen and its degradation products can stimulate the proliferation and migration of smooth muscle cells [30]. These evidences indicate that hyperfibrinogenemia is one of the risk factors for ISR.

A meta-analysis showed that the risk of PAD was higher in patients with familial hypercholesterolemia [31]. Excessive cholesterol in the vascular promotes endothelial dysfunction and activates inflammatory cells, which leads to increased production of inflammatory cytokines and reactive oxygen species, increased expression of adhesion molecules, which is an important step in the development of PAD [32]. Hyperlipidemia plays an equally important role in ISR [33]. Anatomical and intravascular imaging studies have recognized that neoatherosclerosis plays an important role in the occurrence of ISR. Neoatherosclerosis is defined as foamy macrophage infiltration into the peri-strut or neointimal area after stent implantation, potentially leading to ISR, and hyperlipidemia accelerates this process [34, 35]. In addition to hyperlipidemia, the below-the-knee run-offs is an independent risk factor affecting the patency of stents [36], which was also verified in our study. Initiative management of below-the-knee run-offs may reduce the incidence of ISR. Surprisingly, smoking, stent length and stent location were not included in this predictive models, which does not mean they are not risk factors for ISR. On the contrary, it only proves that their predictive value is lower than the above four risk factors.

ISR in the peripheral arteries tends to involve longer and larger-diameter stents. Excessive stent length may cover healthy vessel segments, increasing the risk of in-stent thrombosis and vessel perforation. Insufficient stent length may result in incomplete coverage of the lesion area, leaving stenotic segments and increasing the risk of restenosis. The length of the stent in the ISR group is 150.23 ± 106.91 while the length of the stent in the non-ISR group is 142.24 ± 105.87. The authors'study showed no causal relationship between stent length and ISR, which is consistent with the results of the meta-analysis by Guo et al [37]. However, recent studies have shown that stent length is an independent predictor of ISR after vertebral artery stenting and is positively associated with ISR [38]. In the author's opinion, this may be related to the following factors. These studies focused on the overall stent length difference between the ISR and non-ISR groups and lacked more detailed subgroup analyses. Second, stent diameter as well as stent type may be potential confounders of stent length. It could be investigated whether stent length is related to ISR when stent type as well as stent diameter are the same. Similarly, the relationship between stent diameter and stent type and ISR can be investigated using the method described above, trying to exclude confounding interferences.

In the present study, the association between smoking history and ISR was not significant. The authors speculate that this may be mainly related to the following reasons: the sample was from a single centre, and patient groups susceptible to smoking may not have been included; the limited effect of smoking on ISR after stenting, and the fact that smoking is widely regarded as a risk factor for PAD, and most of the associations between smoking and ISR come from retrospective studies, which are susceptible to bias. Furthermore, this is consistent with the study by Claudio et al., who showed that smoking was not associated with ISR after renal artery stenting [39].

The authors also investigated the relationship between ISR and stent location and showed that there was no significant difference in ISR between the iliac and femoral arteries. Most of the previous studies examining stent location and ISR have been on cranial vessels, particularly the vertebrobasilar artery [40, 41]. Their research showed a higher incidence of ISR at the vertebrobasilar junction (*P* = 0.010), and the incidence of ISR and in-stent ischaemia was higher after intracranial vertebrobasilar stenting than after extracranial procedures (*P* = 0.008 and *P* = 0.002, respectively). The study by Adrien et al. showed a higher incidence of postprocedural not ISR in both the iliac and femoral arteries if the ideal stent selected by preprocedural CTA for precise stenosis lesion selection matched the actual stent selected intraprocedurally on the basis of experience by 2D angiography(iliac: 90% vs. 62.5%, *p* = 0.045; femoral: 77.8% vs. 50%, *p* = 0.057) [42]. However, their study did not show an association between stent position and ISR. In the author's opinion, this may be related to the following points. Firstly, intracranial vascular anatomy is complex and requires high surgical requirements, especially such stenosis as vertebrobasilar junction is more prone to postoperative ISR. The relatively simple anatomy of the iliac and femoral arteries makes them less difficult to operate on, and therefore there is no significant difference in postoperative ISR between them. Second, this study simply classified stent locations into iliac and femoral arteries and did not make a more detailed distinction. In the future, it can be investigated whether there is a difference in the incidence of ISR in different iliac or femoral artery segments.

This study has some limitations. Due to differences in vascular anatomy and haemodynamics, the author speculates that there may be differences between the iliac and femoral regions. However, the manuscript does not make this distinction in detail. Future studies will examine this in more detail based on differences in vascular anatomy and function. Popliteal artery stenting was not selected for this study in terms of the selection of arterial lesion segments. Future studies will fully consider the difference in the occurrence of ISR after stenting of the femoral artery and its important branches and popliteal artery. DCB therapy and stenting are two important interventional therapies in vascular surgery. To avoid potential bias, this paper excluded patients treated with DCB and examined only how the occurrence of ISR differed after iliac and femoral artery stenting. However, for certain complex lesions, such as those where a stent has been inserted but stenosis remains, DCB treatment may be required at this time. This part of the patient population was not included in our study. Future studies could expand the study to also examine the efficacy of treatment with DCB only, stenting only, and treatment with both DCB and stenting. In addition, peak systolic value (PSV) refers to the highest value of blood flow velocity in a blood vessel, usually the maximum blood flow velocity that occurs during cardiac systole. It is measured at the site of a stenosis or lesion and is used to assess the severity of haemodynamics. In patients with stenosis confirmed by postoperative DSA and less than 30%, the author did not use PSV for evaluation. Therefore, this study does not provide the most intuitive indication of the haemodynamic severity of the stenosis site. Meanwhile, our study did not consider the type of stent, such as bare metal stent or drug-eluting stent. The former can provide mechanical support, dilate stenotic vessels and restore blood flow. The latter both restores blood flow and reduces restenosis rates by pharmacologically inhibiting endothelial proliferation. Therefore, this study may have some bias. Finally, only internal validation was performed in this study, and external validation is needed in the future to improve the clinical applicability and generalisability of nomograms.

To summarize, diabetes, hyperlipidemia, hyperfibrinogenemia and below-the-knee run-offs are independent risk factors for ISR within 2 years after iliac or femoral artery stent placement in patients with PAD, and the nomogram risk prediction model established has good prediction performance and high clinical value.

## Data Availability

The datasets used and/or analysed during the current study available from the corresponding author on reasonable request. If you need to ask us for the data of this study, please contact YanRong Zhang, Email: zhangyanrong3320@163.com or XiangDong Liu, Email: xiangdongliu2004@163.com.

## Abbreviations

Name: Variable assignment and description
ISR: In-Stent Restenosis
PAD: Peripheral Arterial Disease
EPV: Event Per Variable
CTA: Computed Tomography Angiography
DSA: Digital Subtraction Angiography
ABI: Ankle-Brachial Index
MRA: Magnetic Resonance Angiography
AGR: Albumin-to-Globulin Ratio
NLR: Neutrophil-to-Lymphocyte Ratio
PLR: Platelet-to-Lymphocyte Ratio
TG: Triglycerides
LDL-C: Low-Density Lipoprotein Cholesterol
AIC: Akaike Information Criterion
ROC: Receiver Operating Characteristic
AUC: Area Under Curve
DCA: Decision Curve Analysis
MCP1: Monocyte Chemoattractant Protein-1
VCAM-1: Vascular Cell Adhesion Molecule-1
PDMP: Platelet-Derived Microparticle
ET-1: Endothelin-1
PSV: Peak systolic value
